# Neurodevelopmental Needs in Young Boys with Duchenne Muscular Dystrophy (DMD): Observations from the Cooperative International Neuromuscular Research Group (CINRG) DMD Natural History Study (DNHS).

**DOI:** 10.1371/currents.md.4cdeb6970e54034db2bc3dfa54b4d987

**Published:** 2018-10-17

**Authors:** Mathula Thangarajh, Christopher F. Spurney, Heather Gordish-Dressman, Paula R. Clemens, Eric P. Hoffman, Craig M. McDonald, Erik K. Henricson, CINRG Investigators

**Affiliations:** Department of Neurology, Children’s National Health System, Washington, D.C.; Department of Cardiology, Children’s National Health System, Washington, D.C., USA; Center for Translational Science, Children’s National Health System, Washington, D.C., USA; Department of Neurology, University of Pittsburgh, Pittsburgh, PA, USA; Pharmaceutical Sciences, State University of Binghamton, NY, USA; Department of Physical Medicine & Rehabilitation, University of California, Davis School of Medicine, Sacramento, CA, USA; Department of Physical Medicine & Rehabilitation, University of California, Davis School of Medicine, Sacramento, CA, USA; Children's Research Institute, Center for Genetic Medicine, Washington D.C., USA

## Abstract

Introduction: Duchenne muscular dystrophy (DMD) is the most common X-linked neuromuscular condition manifested by progressive skeletal muscle weakness, cardiopulmonary involvement and cognitive deficits. Neurodevelopmental symptoms and signs are under-appreciated in this population despite the recognition that cognition has a major impact on quality-of-life. We describe the neurodevelopmental needs in a large cohort of young boys with DMD from the DMD Natural History Study (DNHS). We explore the association between neurodevelopmental needs and DMD mutation location, and with glucocorticoid use.

Methods: We prospectively evaluated 204 participants between ages 4 to less than 9 years of age with DMD as part of a large, longitudinal, international DNHS. We obtained parent- or primary care-giver report of neurodevelopmental needs as part of their study visit. We assessed the relationship between parent/care-giver neurodevelopmental needs and DMD mutation location, and glucocorticoid use.

Results: The neurodevelopmental needs that were most commonly reported included speech delay (33%), mild developmental delay (24%), significant behavioral problems (16.5%), language impairment (14.5%), learning disability (14.5%), attention-deficit hyperactivity disorder (5%) and autism spectrum disorder (3%). Neurodevelopmental needs were more commonly reported by care-givers in those with DMD mutations downstream of exon 51. There was no relationship between care-giver reported neurodevelopmental needs and glucocorticoid use.

Conclusion: Neurodevelopmental needs are highly prevalent in young boys with DMD. Care-givers report higher neurodevelopmental needs when subjects have DMD mutations downstream of exon 51. Early interventions aimed at cognitive health are critical to improve the quality-of-life of individuals with DMD.

Trial Registration: ClinicalTrials.gov NCT00468832

## Introduction

Neurodevelopmental needs are clinically important but often overlooked co-morbidities in Duchenne muscular dystrophy (DMD), the most common X-linked neuromuscular disease that affects 1 in 5000 live births^1^. Autism spectrum^2^^,^^3^, attention-deficit hyperactivity^3^^,^^4^^,^^5^, and obsessive compulsive disorders^3^ are 4 times more common in DMD than in typically developing children. Developmental delay—particularly expressive language delay—is a core neurological symptom in DMD, and was described as early as Duchenne himself 150 years ago^6^^,^^7^. Over the past several years, there has been increasing recognition of the higher-order cognitive skills that are affected in DMD. These include working memory and executive function deficits^8^, academic under-performance^9^, and increased utilization of school resources^10^. As the life-expectancy in DMD has increased tremendously with survival into the third decade of life, it is increasingly important to identify potential strategies for early and improved screening of neurodevelopmental needs in DMD, and to evaluate pragmatic interventions to improve cognitive health^11^. Most importantly, neurodevelopmental disorders and cognitive disabilities have a significant negative impact on the quality-of-life on a daily basis, and affects overall health maintenance.

Dystrophin plays a critical role in brain function^12^. There are several brain-specific dystrophin isoforms, including the full-length dystrophin (dp427), and shorter dystrophin isoforms (dp260, dp140, and dp71) generated by tissue-specific promoters. The following are the unique first exons for the shorter dystrophin isoforms: exon 30 for dp260, exon 45 for dp140, and exon 63 for dp71^13^^,^^14^. In humans, dystrophin is highly expressed by neurons in the cerebellum, the cerebral cortex, and hippocampus. In neurons, dystrophin localizes within the pre-synaptic density (PSD95), a region that is important for dendritic spine formation and synaptic function^15^.

Few investigators suggest a differential vulnerability in the severity of cognitive involvement when DMD mutations are between exons 45-50, suggesting a critical role for the dystrophin dp140 isoform in cognitive function^16^^,^^17^. Not only do individuals with mutations between DMD exons 45-50 have lower intellectual capacity and impaired information processing compared to individuals with DMD mutations in exons 1-44, they also have smaller total brain volume and gray matter volume^18^.

We systematically evaluated the prevalence of neurodevelopmental needs, stratified based on DMD mutation location, in a large prospectively followed cohort of 204 boys between ages 4 to less than 9 years with DMD. These 204 boys were recruited as part of the DMD Natural History Study (DHNS) conducted by the Cooperative International Neuromuscular Research Group (CINRG)^19^. We describe the neurodevelopmental needs in this young cohort at study enrollment as reported by the parent or primary care-giver.

We present data that supports that young boys with DMD have a high prevalence of neurodevelopmental needs as reported by parent or care-giver. Further, boys with DMD mutations between exons 45-50 reported higher cognitive problems. There was no relationship between neurodevelopmental needs and glucocorticoid use. We conclude that there is an unmet, critical medical need in DMD to develop pragmatic solutions for early detection and intervention of neurodevelopmental needs during a window of neurodevelopmental plasticity.

## Methods

The study was conducted in accordance with the Declaration of Helsinki (2000) and the principles of Good Clinical Practice according to the International Conference on Harmonization. All study participants provided written consent prior to study enrollment. Each site has its IRB which approved the study. At Children’s National Health System, the institutional IRB approved the study (#0159)

**Study participants**

The study was conducted in accordance with the World Medical Association Declaration of Helsinki. All study participants provided written consent prior to study enrollment. The study was approved by the ethical review committee at each of the participating sites. Study subjects were recruited from academic institutions within the United States and at other international sites between the years 2006 and 2014. Subjects in this data analysis were part of the DNHS, a 10-year multi-center, international, prospective longitudinal study of DMD (ClinicalTrials.gov NCT00468832). A detailed study description is available as described in McDonald et al.^19^. We chose to evaluate the 204 participants who were between age 4 to less than 9 years in order to identify neurodevelopmental needs in early childhood. Briefly, study participants were evaluated every three months during the first year of study enrollment, every 6 months during the second year of enrollment, and annually thereafter. The assessments performed at each study visit included physical examination, reviews-of-body systems, medication history, muscle strength quantification, functional measurements, and quality-of-life questionnaires completed by the parent or primary care-giver.

**Dystrophin isoform (dp140) assignment**

Information regarding DMD mutation was available in 152 of the 204 participants. The anonymized study participants’ genetic information including exon boundaries of the DMD mutation was reviewed by a genetic counselor. We categorized mutations upstream of DMD exon 44 as dp140+, and those with mutations downstream of DMD exon 51 were categorized as dp140 negative. All subjects whose mutation fell between DMD exon boundaries 45 to 50 were assigned into the dp140 intermediate group. The categorization of study participants is summarized in Figure 1. There were 53 subjects in the dystrophin dp140+ category, 52 subjects in the dystrophin dp140- category, and 48 subjects in the dystrophin dp140 intermediate category.

**Categorization of participants based on DMD exon boundary.**
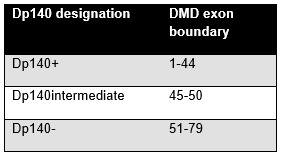

**Evaluation of neurodevelopmental needs**

We evaluated several responses that relate to neurodevelopmental needs from the quality-of-life questions completed by the parent or primary care-giver. These domains focused on twelve neurodevelopmental items, including reports of speech delay, cognitive and learning impairments, and were collected as part of the review of participant medical and developmental history. Reports of study participants attending individual or group therapy, being under-the-care of a psychiatrist or psychologist, and four assessments of school-related activities, (full- or part-time special education, utilization of class-room services, and being on an individualized educational plan (IEP)) were also collected. An IEP refers to the special education, services and additional accommodations that are made available to a child with disability that allow them to attain educational goals and academic milestones. Lastly, pediatric quality-of-life data were captured from all study participants. Here we report the physical, social, emotional, and school sub-scores, along with the total score, from the Pediatric Quality of Life Inventory, version 4 (PedsQoL)^20^. The Pediatric Quality of Life (PedsQL) Inventory is a proxy-report measure designed to measure core health dimensions in children from 5 to 17 years old. The measure consists of 23 items in four scales: physical functioning, emotional functioning, social functioning, and school functioning.

**Statistical Analysis**

Descriptions of the study cohort are provided either as frequencies (N and %) or as summary statistics (N, mean, standard deviation (SD), median, minimum, and maximum) as appropriate for each domain type. Each domain was first compared independently among the three dystrophin dp140 mutation categories. These statistical comparisons were performed using one-way analysis of variance or Kruskal-Wallis tests for continuous outcomes, and Fisher’s exact tests for categorical outcomes. Those neurodevelopmental or cognitive domains showing evidence of a significant relationship with predicted dystrophin dp140 isoform expression were further analyzed using logistic regression. In each logistic regression model, neurodevelopmental or cognitive domain (dichotomous yes/no response) was the dependent variable, and dystrophin dp140 category was the predictive variable. Dystrophin dp140(-) category was considered the reference group, and an additional test comparing the dystrophin dp140(+) and dystrophin dp140 intermediate group was also performed.

In order to assure that neither age nor glucocorticoid use were confounding factors in our data analysis, we compared outcomes between glucocorticoid use (yes/no at baseline visit), and between 1-year age intervals.

For the statistical comparison of responses obtained for the twelve neurodevelopment-related outcomes, we excluded those that answered “Don’t know” or “Not applicable” due to the ambiguity of the response. The rationale to exclude these responses was that we were not able to meaningfully interpret the response, especially for the school-related resources. The “not applicable” response in this setting could be due to the child not attending school, or lack of neurodevelopmental needs, and therefore not needing school services.

All statistical analyses were performed on the data collected at the first study enrollment visit. The significance level for all statistical tests was set at 0.05. Data was analyzed using Stata V15 software (College Station, TX).

## Results

**Study characteristics and demographic information**

A total of 204 participants from North America, Europe, Israel, Australia, and India were included in this young cohort of the DNHS. To determine equity in access to health care, we note that most of the study participants were from developed countries (n=176), whereas 28 participants were from India, a developing country. The mean age of the study participants was 6.4 years (range, 4.0 – 8.9 years). The majority of the study participants identified their race as Caucasian (72.6%), with 17.7% identifying as Asian, 3.5% as Other, 1.0% as Pacific Islander, and 0.5% as Black (0.5%). A minority of the participants (4.9%) reported their race as unknown. Ethnicity was reported as non-Hispanic by 93% of participants and as Hispanic by 7.8% participants. These demographic characteristics, along with anthropometric measures, are summarized in Table 1.

**Table 1:** Summarizes the demographic and anthropometric details of the study participants.

Characteristic	Total cohort	DP140 Negative	DP140 Intermediate	DP140 Positive	P-value comparing DP140 groups
	N (%)	N (%)	N (%)	N (%)	
	Mean ± SD	Mean ± SD	Mean ± SD	Mean ± SD	
	Median (min, max)	Median (min, max)	Median (min, max)	Median (min, max)	
Age (years)	204	52	48	53	0.99
	6.4 ± 1.4	6.5 ± 1.4	6.5 ± 1.3	6.5 ± 1.4	
	6.4 (4.0, 8.9)	6.6 (4.0, 8.9)	6.4 (4.3, 8.9)	6.2 (4.1, 8.9)	
Weight (kg)	204	52	48	53	0.67
	22.0 ± 5.7	22.3 ± 5.9	21.4 ± 6.2	22.4 ± 5.7	
	20.7 (13.4, 48.2)	21.0 (13.4, 37.1)	20.0 (15.0, 48.2)	21.8 (13.5, 41.0)	
Calculated height (cm)	203*	51	48	53	0.85
	116.6 ± 9.2	116.6 ± 9.2	117.0 ± 8.9	116.0 ± 9.6	
	115.7 (95.4, 139.9)	117,8 (96.9, 135.4)	116.3 (99.9, 139.9)	114.9 (95.4, 134.3)	
Race					0.25
Caucasian	148 (72.6%)	39 (75.0%)	31 (64.5%)	39 (73.6%)	
Black	1 (0.5%)	0 (0.0%)	1 (2.1%)	0 (0.0%)	
Pacific Isl.	2 (1.0%)	0 (0.0%)	0 (0.0%)	2 (3.8%)	
Asian	36 (17.7%)	11 (21.2%)	13 (27.1%)	6 (11.3%)	
Other	7 (3.4%)	1 (1.9%)	1 (2.1%)	4 (7.6%)	
Unknown	10 (4.9%)	1 (1.9%)	2 (4.2%)	2 (3.8%)	
Ethnicity					0.22
Non-Hispanic	188 (93.2%)	47 (90.4%)	45 (94.8%)	52 (98.1%)	
Hispanic	16 (7.8%)	5 (9.6%)	3 (6.3%)	1 (1.9%)	

*Height was not reported in one participant during the first year of study.

**Study subjects and their treatment with oral glucocorticoids**

Sixty-one percent of participants (n=124) were on oral glucocorticoids at the time of their baseline study visit (Table 2). Of these 124 participants, 79 participants (39%) were either on prednisone or prednisolone, and 44 participants (22%) were on deflazacort. One participant reported glucocorticoid use but did not indicate the name of the medication being prescribed. The duration of cumulative use of glucocorticoid ranged from no previous use to 4.5 years.

**Table 2:** Summarizes the glucocorticoid use in study participants.

	Total cohort	DP140 Negative	DP140 Intermediate	DP140 Positive	P-value comparing DP140 groups
	N (%)	N (%)	N (%)	N (%)	
	Mean ± SD	Mean ± SD	Mean ± SD	Mean ± SD	
	Median (min, max)	Median (min, max)	Median (min, max)	Median (min, max)	
Is the study participant taking steroids at baseline					0.39
Yes	124 (60.8%)	29 (55.8%)	33 (68.8%)	34 (64.2%)	
No	80 (39.2%)	23 (44.2%)	15 (31.3%)	19 (35.9%)	
Total lifetime steroid use at baseline (days)	204	52	48	53	0.34
	288 ± 382	291 ± 390	241 ± 340	355 ± 423	
	95 (0, 1665)	53 (0, 1182)	95 (0, 1665)	208 (0, 1579)	
Type of steroid taken*					0.92
None	80 (39.4%)	23 (44.3%)	15 (31.9%)	19 (35.9%)	
Prednisone	48 (23.7%)	12 (23.1%)	13 (27.7%)	15 (28.3%)	
Deflazacort	44 (21.7%)	11 (21.2%)	11 (23.4%)	11 (20.8%)	
Prednisolone	31 (15.3%)	6 (11.5%)	8 (17.0%)	8 (15.1%)	

**Care-giver reported neurodevelopmental needs**

The response completion of neurodevelopmental needs identified by the care-giver was variable depending on the question, ranging from 168 to 174 respondents (82 - 85%) of the 204 participants at the first study visit. The following neurodevelopmental needs were most commonly reported: speech delay (33%), mild developmental delay (24%), significant behavioral problems (16.5%), language impairment (14.5%), and learning disability (14%). Other neurodevelopmental needs reported by the parent or primary care-giver included attention-deficit hyperactivity disorder (5%) and autism spectrum disorder (3%). Summaries of responses from the entire cohort are shown in Supplementary Table 1.

With regards to therapy services, most respondents had not received any (Supplemental Table 2). With regards to utilization of school-based resources at the first study visit, of the 169 respondents, an equal number of participants were on an IEP (n= 65) as not on an IEP (n=65) (Supplemental Table 3). Thirty-six participants (22%) reported that an IEP was not applicable in their child’s case, though thirteen of these participants (36%) were under the age of 5 years. The commencement of school is age 5 years in developed countries. These thirty-six participants were excluded from the subsequent analyses since no further information was available from them. Sixty-three percent (n=106) reported receiving no services in the school compared to 27% (n=46) receiving classroom services (Supplemental Table 3). Of those not receiving classroom services, 10% (n=17) responded that they were not applicable and 1% (n=2) did not know whether their child was receiving any services. Fourteen of the “no responses” and 10 of the “not applicable” responses were in participants under the age of 5 years.

When participants were asked whether they had been evaluated by a mental health provider (psychiatrist or psychologist), most of them participants had not been (Supplemental Tables 4, 5).

When study participants were stratified based on 1-year age intervals at study enrollment, parent or primary care-giver of participants identified between boys between 7 to 8 years of age as having more behavioral, neurodevelopmental and cognitive needs (significant behavioral problems, speech delay, language impairment, learning disability) (Table 3). Similarly, IEP and utilization of services in the classroom were more often reported in participants 7 years to 8 years of age (Table 4). Of the 169 respondents, 141 (83%) report that they had never been evaluated by a psychologist.

**Table 3:** Summarizes neurodevelopmental challenges based on age groups.

Have you ever been diagnosed with-	Age group	No	Yes	Don't know	P-value*
Significant behavioral problems	4 to <5	25	2	1	0.59
	5 to <6	36	6	2	
	6 to <7	27	6	0	
	7 to <8	29	8	1	
	8 to <9	25	6	0	
Depression	4 to <5	27	0	1	0.74
	5 to <6	44	0	0	
	6 to <7	33	0	0	
	7 to <8	36	1	1	
	8 to <9	31	0	0	
Autism	4 to <5	27	1	0	0.21
	5 to <6	42	0	2	
	6 to <7	32	1	0	
	7 to <8	35	3	0	
	8 to <9	30	0	0	
Speech delay	4 to <5	16	12	0	0.15
	5 to <6	28	13	3	
	6 to <7	24	9	0	
	7 to <8	21	17	0	
	8 to <9	25	6	0	
Language impairment	4 to <5	23	4	1	0.20
	5 to <6	37	4	3	
	6 to <7	26	4	3	
	7 to <8	28	10	0	
	8 to <9	28	2	1	
Learning disability	4 to <5	26	2	0	0.018
	5 to <6	38	3	3	
	6 to <7	27	3	3	
	7 to <8	25	12	1	
	8 to <9	28	3	0	
Sensory integration disorder	4 to <5	27	1	0	0.51
	5 to <6	40	1	3	
	6 to <7	32	0	1	
	7 to <8	33	3	2	
	8 to <9	30	1	0	
Cognitive impairment	4 to <5	26	2	0	0.31
	5 to <6	36	4	1	
	6 to <7	31	0	2	
	7 to <8	32	4	2	
	8 to <9	29	1	1	
ADHD	4 to <5	27	0	1	0.28
	5 to <6	42	1	1	
	6 to <7	31	1	1	
	7 to <8	32	4	2	
	8 to <9	27	2	2	
Mental retardation	4 to <5	27	1	0	0.59
	5 to <6	42	2	0	
	6 to <7	33	0	0	
	7 to <8	37	0	1	
	8 to <9	30	1	0	
Mild developmental delay	4 to <5	17	10	1	0.24
	5 to <6	32	10	1	
	6 to <7	29	4	0	
	7 to <8	27	10	0	
	8 to <9	23	6	2	
Pervasive developmental disability	4 to <5	27	0	1	0.14
	5 to <6	42	0	1	
	6 to <7	32	0	0	
	7 to <8	34	2	2	
	8 to <9	28	0	2	

*P-value calculated using total who answered yes or no (excludes those indicating they don’t know)

**Table 4.** Summarizes the use of services based on age groups.

Outcome	Outcome response - No	Outcome response - Yes	Age group	OR	P-value	95% CI
Classroom services	14	3	4 to <5	1.00		
	26	11	5 to <6	1.97	0.35	0.47 – 8.27
	26	7	6 to <7	1.26	0.77	0.28 – 5.63
	15	20	7 to <8	6.22	0.011	1.51 – 25.62
	25	5	8 to <9	0.93	0.93	0.19 – 4.50
Individual therapy	27	1	4 to <5	1.00		
	42	0	5 to <6	---	---	
	31	2	6 to <7	1.74	0.66	0.15 – 20.29
	33	4	7 to <8	3.27	0.30	0.35 – 31.04
	30	1	8 to <9	0.90	0.94	0.05 – 15.10
Group therapy	27	1	4 to <5	1.00		
	41	1	5 to <6	0.66	0.77	0.04 – 10.98
	32	1	6 to <7	0.84	0.91	0.05 – 14.13
	35	3	7 to <8	2.31	0.48	0.23 – 23.51
	30	1	8 to <9	0.90	0.94	0.05 – 15.10
Seeing a psychologist	25	3	4 to <5	1.00		
	40	2	5 to <6	0.42	0.36	0.06 – 2.67
	25	7	6 to <7	2.33	0.27	0.54 – 10.06
	29	8	7 to <8	2.30	0.25	0.55 – 9.61
	22	8	8 to <9	3.03	0.14	0.71 – 12.85

**Glucocorticoid use and reported neurodevelopmental needs**

Of the 204 participants, 124 participants (60.8%) were on oral glucocorticoid. When cognitive domains were compared between glucocorticoid users and glucocorticoid non-users, there was no relationship between glucocorticoid use and any of the cognitive domains (Table 5). The utilization of classroom services or mental health professionals was also not significantly different between these glucocorticoid users and glucocorticoid non-users (data not shown). In addition, no significant difference in the total quality-of-life score or sub-scores was detected between these two groups (data not shown).

**Table 5.** Summarizes the relationship between neurodevelopmental and behavioral challenges and glucocorticoid use.

Have you ever been diagnosed with-	Cumulative lifetime steroid use	No	Yes	Don't know	P-value*
Significant behavioral problems	<6 months	80	11	4	0.15
	≥ 6 months	62	17	0	
Depression	<6 months	94	0	1	0.45
	≥ 6 months	77	1	1	
Autism	<6 months	89	3	2	0.99
	≥ 6 months	77	2	0	
Speech delay	<6 months	66	27	2	0.20
	≥ 6 months	48	30	1	
Language impairment	<6 months	79	11	5	0.39
	≥ 6 months	63	13	3	
Learning disability	<6 months	78	10	7	0.38
	≥ 6 months	66	13	0	
Sensory integration disorder	<6 months	89	2	4	0.41
	≥ 6 months	73	4	2	
Cognitive impairment	<6 months	83	7	3	0.76
	≥ 6 months	71	4	3	
ADHD	<6 months	88	3	4	0.47
	≥ 6 months	71	5	3	
Mental retardation	<6 months	92	3	0	0.63
	≥ 6 months	77	1	1	
Mild developmental delay	<6 months	68	22	4	0.86
	≥ 6 months	60	18	0	
Pervasive developmental disability	<6 months	89	0	4	0.21
	≥ 6 months	74	2	3	

*P-value calculated using total who answered yes or no (excludes those indicating they don’t know)

**Neurodevelopmental needs based on *DMD* mutation location**

We compared the reported cognitive domains affected in the three subcategories of DMD study participants (dystrophin dp140(+), dystrophin dp140(-), and dystrophin dp140 intermediate). Several statistical associations were noted between the three subcategories of study participants, with differences between subcategories in domains of cognitive and language impairments, learning disability, and utilization of classroom services.

For those cognitive domains that showed a statistically significant relationship with DMD mutations affecting the dystrophin dp140 isoform (exon 45-50), further analysis using logistic regression showed that study participants in the dystrophin dp140 intermediate category were more alike those in the dystrophin dp140(+) category (Table 6). Most of the statistical differences detected were between the dystrophin dp140 intermediate and dystrophin dp140(-) categories.

**Table 6.** Summarizes the cognitive outcomes based on the three DMD genotype subgroups.

Cognitive outcome	Mutation	Response - No	Response - Yes	OR	p-value	95% CI	P-value comparing Dp140Intermediate to Dp140Negative
Behavior problems	Negative	34	10	1.00			0.21
	Intermediate	37	3	0.28	0.07	0.07–1.09	
	Positive	40	8	0.68	0.47	0.24–1.91	
Language impairment	Negative	31	11	1.00			0.59
	Intermediate	38	3	0.22	0.031	0.06–0.87	
	Positive	42	5	0.34	0.06	0.11–1.06	
Learning disability	Negative	33	10	1.00			0.26
	Intermediate	38	1	0.09	0.023	0.01–0.71	
	Positive	42	4	0.31	0.07	0.09–1.09	
Classroom services	Negative	17	19	1.00			0.49
	Intermediate	32	6	0.17	0.001	0.06–0.50	
	Positive	36	10	0.25	0.004	0.10–0.65	
Cognitive impairment	Negative	36	7	1.00			---
	Intermediate	39	0	---	---		
	Positive	44	2	0.24	0.08	0.05–1.20	

Dystrophin dp140 intermediate participants were significantly less likely to report affected cognitive domains than dystrophin dp140(-) participants. In the domains of cognitive impairment and behavioral problems, there was no statistical significance between the three groups. Participants in the dystrophin dp140 intermediate group were significantly less likely to have a diagnosis of significant language impairment (OR=0.22, 95% CI 0.06 – 0.87) and learning disability (OR=0.09; 95% CI 0.01 – 0.71). Further, a statistically significant relationship between the utilization of classroom services and DMD mutation subcategory, with both the dystrophin dp140(+) and dystrophin dp140 intermediate categories being less likely to utilize classroom services than those who were dystrophin dp140(-) (OR=0.17, 95%CI 0.06-0.5; OR=0.25, 95%CI 0.10-0.65, respectively). There were no statistically significant differences between the dystrophin dp140(+) and dystrophin dp140 intermediate categories in any of the cognitive domains. There were also no statistically significant differences in the utilization of mental health professionals or quality-of-life assessments between the three subcategories (data not shown).

**Quality-of-life assessment**

Quality-of-life was assessed using the PedQoL questionnaire and a total of 141 (69%) care-givers completed the questionnaire. One questionnaire was completed per participant. Mean (±SD) sub-scores for physical, emotional, social, and school domains were all below 50 (41.9 ± 21.2, 25.4 ± 18.3, 34.2 ± 10.5, and 29.2 ± 20.0, respectively). The mean total score for all 141 participants was 33.9 ± 16.0 and ranged from 4.4 to 70.7 (Supplemental Table 6). None of the participant in the study scored the maximum score of 100.

## Discussion

In this report, we present data as assessed by the DNHS to support the prevalence of neurodevelopmental needs in young boys with DMD. To our knowledge, this is the first prospectively collected data of neurodevelopmental needs from a large well-characterized, internationally representative cohort of young boys with DMD. We also stratified boys with DMD mutations into three sub-categories based on DMD mutations likely to affect the dystrophin dp140 isoform to characterize in greater detail the neurodevelopmental needs in this cohort. By contrast, both Felisari et al.^16^ and Bardoni et al.^17^ evaluated cognitive impairment based on assessment of intellectual capacity through intelligence quotient, while our data is based on parent- or primary care-giver report.

Speech delay was the most commonly reported neurodevelopmental need in our cohort. Speech delay was reported by 33% of our respondents, and is consistent with reports of higher prevalence of speech delay in DMD^7^^,^^10^. Speech delay is a predictor of later education and classroom services utilization. Soim et al. describe that receiving speech therapy is statistically associated with an increased prevalence of grade repetition^10^. Thus, speech delay in DMD has implications in the developmental trajectory of affected boys, and suggests that these children may need additional resources to achieve optimal educational outcomes. Such additional resources include earlier psycho-educational assessment in boys with DMD identified with speech delay. Recognition of early learning differences would potentially facilitate establishing appropriate school support and prevent grade repetition. Not only would it improve the quality of school experience for these children, it would also help improve long-term educational outcomes. Our collective experience as professionals engaged in the care of DMD families, psycho-educational testing often times, does not occur until age 7 or 8 years, thus delaying the care-givers and educators awareness of the unique neurodevelopmental needs in young boys with DMD. This lack of awareness may be the reason that parent-reported concerns were particularly more frequent in boys between the ages of 7 to less than 8 years in our cohort.

The reported prevalence of attention-deficit and autism that is described in our cohort is different from those reported from earlier studies. For example, Ricotti et al. evaluating boys with DMD of ages 5 to 17 years noted that the prevalence of autism spectrum was 21%^21^. They used both a neurodevelopmental questionnaire and a structured diagnostic interview. Pane et al. found that nearly 32% of boys with DMD fulfilled criteria for attention deficit-hyperactivity using DSM-IV criteria. The mean age of their cohort was 12.6 years^4^. Thus, the differences reported in our cohort is likely methodological in nature. Furthermore, the mean age of our cohort was 6.4 years, and full manifestations of attentional challenges and hyperactivity may not be fully manifest.

To our knowledge, there is limited data on the long-term educational and employment impact of cognitive needs in DMD. We note that parent-reported concerns were particularly frequent in boys between ages 7 to less than 8 years (Tables 4 and 5). Most of the study participants in our data are from developed countries, and age at school entry is approximately 5 years. We reason that as they transition from pre-school to primary school, neurodevelopmental needs may become increasingly apparent. This may be in addition possibly to the lack of awareness of the unique neurodevelopmental needs in this population by care-givers and educators.

School and emotional sub-scores scored among the lowest average sub-scores with PedsQoL, demonstrating that these two sub-domains may be most affected by disease burden in this cohort (Supplemental Table 6). While the exact reasons for these findings are unclear, our data suggests that neurodevelopmental and behavioral needs may influence school-related experiences in young children. Further detailed analyses in this area are warranted to understand the impact of neurodevelopmental needs in educational outcomes.

The use of glucocorticoids has been shown to be associated with behavioral and mood changes^22^. In this young cohort of boys with DMD, we found that approximately 60% of them were on oral glucocorticoids at the time of entry to study. Consistent with other reports^23^^,^^24^, we did not find a statistically significant association between reported neurodevelopmental concerns and glucocorticoid use. Population-based longitudinal data does support that evaluated longitudinally, glucocorticoid use is associated with behavioral difficulties in older boys with DMD^5^. The cross-sectional nature of our data analysis and the younger age of our cohort may explain this difference with existent data.

Clinical and genetic heterogeneity in DMD has been well-described^25^^,^^26^^,^^27^ and it has previously been noted that cognitive outcomes may predict motor, cardiac and respiratory outcomes in DMD^28^. Boys with DMD who had poor cognition, also had worse cardiac outcome, with two-thirds of these boys having cardiac involvement before age 12. This data suggests that overall health maintenance in DMD may be dictated in part by cognitive health, or a genetic modifier, or both.

Our study has some limitations. First, the DNHS was not designed to assess in detail the neurodevelopmental needs of this cohort. Therefore, there was no collection of objective psychometric data during the study. Existent data does suggest that neurodevelopmental needs of DMD are present even during infancy^29^. Chieffo et al. report that young boys with DMD showed clear developmental concerns when followed prospectively^30^.

The second limitation of our study is that some of the recorded responses were ambiguous, and some parent- or care-giver reported concerns have overlapping features. For example, there was lack of distinction between “cognitive and language impairment” on the questionnaire. Neurodevelopmental needs may exist on a continuum and may overlap; for example, there may be co-existent speech delay and language impairment. Particularly in DMD, two or more neurodevelopmental disorders can co-exist, highlighting the overlap in developmental pathways affected. Despite these limitations, our data highlights the clear neurodevelopmental burden in this medically vulnerable population. Our data suggests that future studies in DMD should consider the use of standardized reports such as patient-reported outcome measures or the muscular dystrophy quality-of-life questionnaire. Further, specific recommendations in future studies in DMD should consider standardized assessment of cognitive ability.

Taylor et al. reported that the cumulative loss of multiple brain-specific dystrophin-isoforms may increase the burden of neurocognitive deficits in DMD^31^. The existence of multiple brain-specific dystrophin isoform suggests a redundancy in some of its functions in the developing brain. We chose to particularly focus the analysis in DMD region exon 45-50 based on the current clinical and research priority to understand better the heterogeneity within this sub-group based on the available exon 51 skipping therapy, and recent published report on the structural and functional compromise to the developing brain due to DMD mutations that affect dystrophin dp140 isoform^18^.

Our data highlights a critical, yet often unmet medical need in this population. It is a widely held notion that cognitive impairments are irreversible in neurodevelopmental disorders. This notion is being challenged by the emergence of new scientific data that modulation of receptors can result in improvement of cognitive function^32^. Our data provides a focal point on how future studies in DMD should aim to address neurodevelopmental needs more comprehensively through collection of objective psychometric data longitudinally. This approach will provide important scientific data to enable development of pragmatic interventions to improve educational and workplace integration for individuals with DMD, many of whom now have the capacity to attain independency as adults in society.

## Supporting Information

**S1 Table:** Description of cognitive outcomes at the baseline visit.

Have you ever been diagnosed with…	No*	Yes	Don't Know	Total answered
Significant behavioral problems	142 (83.5%)	28 (16.5%)	4	174
Depression	171 (99.4%)	1 (0.6%)	2	174
Autism	166 (97.1%)	5 (2.9%)	2	173
Speech delay	114 (66.8%)	57 (33.3%)	3	174
Language impairment	142 (85.5%)	24 (14.5%)	8	174
Learning disability	144 (86.2%)	23 (13.8%)	7	174
Sensory integration disorder	162 (96.4%)	6 (3.6%)	6	174
Cognitive impairment	154 (93.3%)	11 (6.7%)	6	171
ADHD	159 (95.2%)	8 (4.8%)	7	174
Mental retardation	169 (97.7%)	4 (2.3%)	1	174
Mild developmental delay	128 (76.2%)	40 (23.8%)	4	172
Pervasive developmental disability	163 (98.8%)	2 (1.2%)	6	171

*Percentages calculated using total who answered yes or no (excludes those indicating they don’t know).

**S2 Table:** Description of therapy outcomes at the baseline visit.

Have you ever had…	No	In the past	Occasionally	Daily or regularly	Total answered
Individual therapy	163 (95.3%)	3 (1.8%)	2 (1.2%)	3 (1.8%)	171
Group therapy	165 (95.9%)	4 (2.3%)	1 (0.6%)	2 (1.2%)	172

**S3 Table:** Description of school related outcomes at the baseline visit.

Have you ever had…	No*	Yes	Not applicable	Don't know	Total answered
IEP or 504 plan	65 (39.2%)	65 (39.2%)	36 (21.7%)	3	169
Services in the classroom	106 (62.7%)	46 (27.2%)	17 (10.1%)	2	171
Part-time special education	119 (71.3%)	27 (16.2%)	21 (12.6%)	4	171
Full-time special education	135 (81.8%)	8 (4.9%)	22 (13.3%)	5	170

*Percentages calculated using total who answered yes, no, or not applicable (excludes those indicating they don’t know)

**S4 Table:** Description of psychiatry use at the baseline visit.

Have you ever seen a…	No	Yes	Total answered
Psychiatrist	158 (94.1%)	10 (6.0%)	168

**S5 Table:** Description of psychology use at the baseline visit.

Have you ever seen a…	Never	Weekly	Monthly	Quarterly	As needed	Total answered
Psychologist	141 (83.4%)	2 (1.2%)	2 (1.2%)	4 (2.4%)	20 (11.8%)	169

**S6 Table:** Description of PedsQL assessments at the baseline visit.

PedsQOL score	N	Mean ± SD	Median (min, max)
Physical sub-score	141	41.9 ± 21.2	40.6 (0, 100)
Emotional sub-score	141	25.4 ± 18.3	25 (0, 75)
Social sub-score	141	34.3 ± 10.5	35 (0, 100)
School sub-score	133	29.2 ± 20.0	25 (0, 80)
Total score	141	33.9 ± 16.0	32.6 (4.4, 70.7)

*Percentages calculated using total who answered yes or no (excludes those indicating they don’t know)

## Funding

DNHS was funded through grants from the U.S. Department of Education/NIDRR (H133B031118 and H133B090001), U.S. Department of Defense (W81XWH-09-1-0592), the National Institutes of Health (UL1RR031988, U54HD053177, UL1RR024992, U54RR026139, G12RR003051, 1R01AR061875, and RO1AR062380), and Parent Project Muscular Dystrophy.

Data analysis for this manuscript was supported by Award Number UL1TR001876 from the NIH National Center for Advancing Translational Sciences to C.S. Its contents are solely the responsibility of the authors and do not necessarily represent the official views of the National Center for Advancing Translational Sciences or the National Institutes of Health.

Mathula Thangarajh was supported the American Brain Foundation/American Academy of Neurology Clinical Research Fellowship (2015 - 2017), and also gratefully acknowledges the support by National Institutes of Health grant (R25NS088248-02; Principal Investigator Dr. Meurer).

The funders had no role in study design, data collection and analysis, decision to publish, or preparation of the manuscript. The authors take full responsibility for the contents of the manuscript. The views presented in this report do not represent the views of the Department of Defense or the U.S. Government.

## Competing Interests

Mathula Thangarajh has provided consultant services to PTC Therapeutics. Christopher Spurney has no conflict of interest to disclose. Heather Gordish-Dressman has served as a consultant for AGADA BioSciences and Solid GT, and is a co-founder and part owner of TRiNDS. Paula R Clemens serves on data safety monitoring boards for Pﬁzer and NIH and has served as a consultant to NS Pharma and Sanoﬁ/Genzyme. She currently receives grant support from NS Pharma, Sanoﬁ/Genzyme, Amicus, MDA, Department of Defense, and NIH. She is board member for TRiNDS. Eric P Hoffman has served on advisory committees for AVI BioPharma, as a consultant with AGADA BioSciences, the Gerson Lehrman Group, Medacorp, and Lazard Capital, is cofounder, board member, and shareholder of ReveraGen, is a co-founder and part owner of TRiNDS, and has a patent pending related to the treatment of Duchenne muscular dystrophy. Craig McDonald has served as a consultant for clinical trials for PTC Therapeutics, BioMarin, Sarepta, Eli Lilly, Pﬁzer, Halo Therapeutics, Santhera Pharmaceuticals, Cardero Therapeutics, Catabasis, Marathon, and Mitokyne, outside the submitted work; serves on external advisory boards related to Duchenne muscular dystrophy for PTC Therapeutics, Eli Lilly, Sarepta, Mitokyne, and Marathon; and reports grants from the US Department of Education/National Institute on Disability and Rehabilitation Research (NIDRR), National Institute on Disability, Independent Living, and Rehabilitation Research (NIDILRR), US National Institutes of Health (NIH)/National Institute of Arthritis and Musculoskeletal and Skin Diseases (NIAMS), US Department of Defense, and US Parent Project Muscular Dystrophy, during the conduct of the study. Erik Hendricson has served as a consultant for Santhera Pharmaceuticals, Cardero Therapeutics, Bristol-Myers Squibb, Genzyme, and PTC Therapeutics, and has served as an external advisory board member for Parent Project Muscular Dystrophy and as an executive committee member of the Cooperative International Neuromuscular Research Group. RTA has served as a consultant for Santhera Pharmaceuticals. There are no patents, products in development or marketed products to declare. The competing interests do not alter the authors' adherence to all the PLOS policies on sharing data and materials.

## Data Availability

Data presented in this manuscript was collected as part of the DMD Natural History Study (DNHS). The CINRG executive committee has restrictions on who accesses the data, as there are many datasets within this study. Data requests can be made to Lauren Morgenroth ‎[lmorgenroth@trinds.com].

## Corresponding Author

Mathula Thangarajh, MD, PhD

Department of Neurology

Children's National Health Systems

Washington, D.C. 20010

Phone: 202-476-2771

Fax: 202-476-2864

Email: mthangar@childrensnational.org
